# Suhuang antitussive capsule at lower doses attenuates airway hyperresponsiveness, inflammation, and remodeling in a murine model of chronic asthma

**DOI:** 10.1038/srep21515

**Published:** 2016-02-10

**Authors:** Chao Zhang, Lan-Hong Zhang, Yin-Fang Wu, Tian-Wen Lai, Hai-Sheng Wang, Hui Xiao, Luan-Qing Che, Song-Min Ying, Wen Li, Zhi-Hua Chen, Hua-Hao Shen

**Affiliations:** 1 Department of Respiratory and Critical Care Medicine, Second Affiliated Hospital, Institute of Respiratory Diseases, Zhejiang University School of Medicine, Hangzhou, China; 2 Yangtze River Pharmaceutical Group Beijing Haiyan Pharmaceutical Co., Ltd, Beijing, China.; 3 State Key Lab of Respiratory Disease, Guangzhou, China.

## Abstract

Suhuang antitussive capsule (Suhuang), a traditional Chinese medication, is found effective in treating chronic cough and cough variant asthma (CVA). This study aimed to determine the possible effects and underlying mechanisms of Suhuang on chronic ovalbumin (OVA)-induced airway hyperresponsiveness (AHR), inflammation, and remodeling in mice. Mice were randomly assigned to six experimental groups: control, OVA model with or without Suhuang (low dose: 3.5 g/kg, middle dose: 7.0 g/kg, high dose: 14.0 g/kg), or dexamethasone (2.5 mg/kg). AHR, inflammatory cells, cytokines in bronchoalveolar lavage fluid (BALF), lung pathology, mucus production, and airway remodeling were examined. We found Suhuang treated at lower doses effectively inhibited OVA-induced AHR, airway inflammation, mucus production and collagen deposition around the airway. High dose of Suhuang reduced most of the inflammatory hallmarks while exerted inconsiderable effects on the number of macrophages in BALF and AHR. At all doses, Suhuang significantly reduced the levels of interlukin (IL) -13 and transforming growth factor (TGF)-β1, but had little effects on IL-4, IL-5, IL-17A and interferon (IFN)-γ. Thus, Suhuang administration alleviates the pathological changes of chronic asthma likely through inhibition of IL-13 and TGF-β1. Suhuang might be a promising therapy for patients with allergic asthma in the future.

Asthma is one of the most common chronic respiratory diseases, which is characterized by the persistence of chronic airway inflammation, airway hyperresponsiveness (AHR), and mucus hypersecretion^1^. The allergic airway inflammation, in general, is comprised of a predominant T helper (Th) 2 immune response and subsequently produced eosinophils^1^,^2^. The persistent inflammation eventually leads to airway remodeling, which includes a variety of structural changes in the epithelium and other portions of the airway, such as airway wall thickening, epithelial hypertrophy, mucus metaplasia, subepithelial fibrosis, smooth muscle hyperplasia and hypertrophy, frequently seen in asthma individuals^3^. Airway remodeling induced by infectious or allergic factors leads to airway narrowing and limitation, which is related to the persistence and severity of asthma^4^.

Asthma imposes high social and economic costs. Furthermore, disease outcomes remain suboptimal despite current effective treatment modalities such as inhaled corticosteroids (ICS). The use of complementary or alternative medicine (CAM) in asthma patients is increasing as an adjunct and also as a substitute for effective and proven anti-inflammatory therapies^5^,^6^. Herbal remedies are a consistently popular form of CAM in asthma, and proprietary asthma drugs are derived from herbal remedies-for example, tealeaves are the herbal origin of theophylline^5^,^7^. It has been reported that 11–40% of people with asthma use herbal remedies^5^,^8^,^9^.

Suhuang antitussive capsule (Suhuang) is a traditional Chinese patent drug widely used for treating chronic cough and cough variant asthma (CVA), which is composed of 9 traditional Chinese herbs (details in Methods below): *Folium perillae, Herba ephedrae, Pheretima, Periostracum cicadae, Fructus arctii, Fructus schisandrae chinensis, Folium eriobotryae, Radix peucedani* and *Fructus perillae.* This drug has been shown to suppress inflammation, to increase activity of reticular endothelial system, to up-regulate T lymphocyte subsets, and to improve the immune function in patients with CVA^10^,^11^,^12^. Suhuang has also been reported to have an obvious effectiveness and safety in treating cough of chronic obstructive pulmonary disease (COPD) stable stage with the low recurrence rate after medication discontinuation^13^. Therefore, Suhuang may have potential efficacy in controlling inflammatory diseases. However, the possible benefits of Suhuang in asthma therapy have not yet been thoroughly investigated. The present study therefore attempted to explore the role of Suhuang on lung histopathology in a murine model of asthma, and to investigate its possible underlying molecular mechanisms.

## Results

### Suhuang prevented AHR in chronic OVA-challenged mice at lower doses

**Figure 1:**
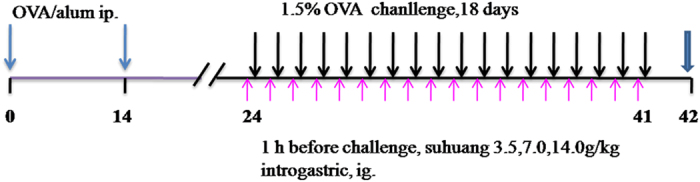
Time line representation of the chronic asthma model and pharmacological intervention.

**Figure 2:**
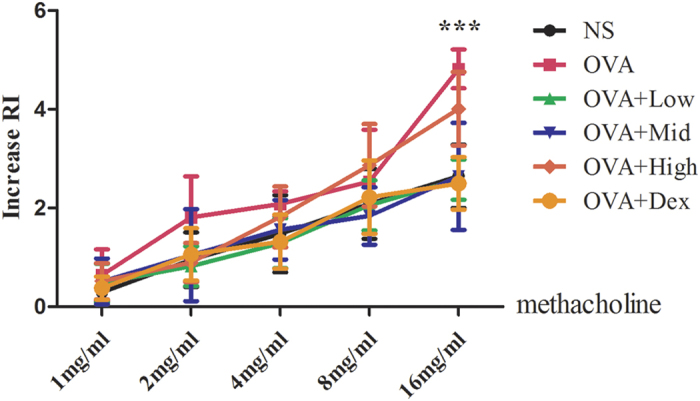
Suhuang attenuates AHR in OVA-challenged mice at lower doses. NS: saline controls; OVA + Low: OVA challenge and treated with 3.5 g/kg Suhuang; OVA + Mid: OVA challenge and treated with 7.0 g/kg Suhuang; OVA + High: OVA challenge and treated with 14.0 g/kg Suhuang; OVA + Dex: OVA challenge and treated with 2.5 mg/ml dexamethasone. Data were shown as mean ± SEM (***p < 0.001).

AHR is the most important pathological feature of asthma, and is always used to distinguish asthma from other airway inflammatory diseases. We first determined whether Suhuang treatment could improve AHR of allergic mice after chronic OVA challenge (Fig. 1). An invasive method was undertaken to determine the increased airway resistance (RI) by methacholine (Mch). Results revealed that AHR was significantly increased in the OVA group when compared with normal saline (NS) controls (Fig. 2). Suhuang treatment at low and middle doses significantly decreased AHR, especially at a dose of 16 mg/ml Mch (NS vs OVA, p < 0.001; OVA vs low dose, p < 0.001; OVA vs middle dose, p < 0.001). There were no significant differences between the dexamethasone group and groups treated with either low or middle doses of Suhuang. At high dose, however, Suhuang treatment was only slightly but not significantly decreased AHR relative to the OVA group (Fig. 2).

### Suhuang alleviated OVA-induced eosinophilic airway inflammation

**Figure 3:**
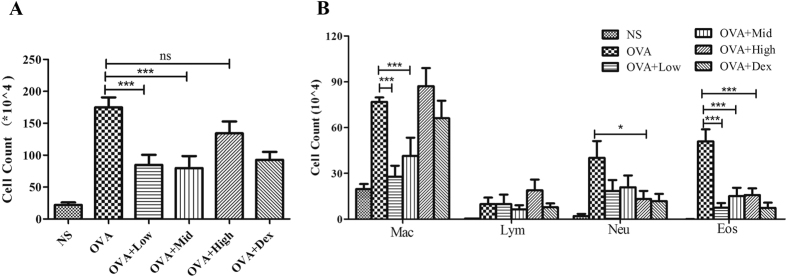
Suhuang ameliorates allergic airway inflammation in the BALF. (**A**) Total cell count in BALF. (**B**) Cell differentation of BALF. Groups are labeled as in legends of Fig. 2. Mac: macrophages; Lym: lymphocytes; Neu: neutrophils; Eos: eosinophils. Data were shown as mean ± SEM (ns: no significant; *p < 0.05; ***p < 0.001).

To determine whether Suhuang could exert any considerable effects on allergic airway inflammation, all mice from each group were sacrificed to collect the BALF. Total cell count indicated that low and middle doses of Suhuang administration significantly alleviated OVA induced inflammation (p < 0.001, Fig. 3A), while Suhuang at high dose exhibited a less protective effect (Fig. 3A). However, Suhuang at all three doses markedly decreased the OVA-induced eosinophils in BALF (Fig. 3B). Neutrophils were also notably decreased by Suhuang at either dose. (Fig. 3B). For macrophages, only low and middle doses of Suhuang significantly reduced the number, while high dose of Suhuang failed to affect it, which is consistent with the total BALF cell count (Fig. 3A). Lymphocytes appeared not to be affected by Suhuang at all doses (Fig. 3B).

**Figure 4:**
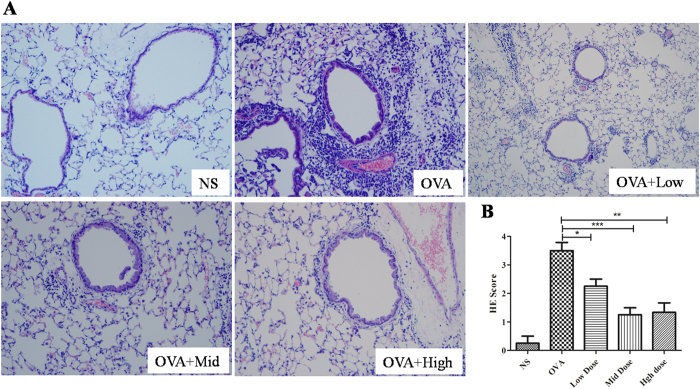
Suhuang alleviates inflammatory infiltration in lung tissues. (**A**) Representive images of H&E staining sections of pulmonary inflammation of each group. (**B**) Score of peribronchiolar and perivascular inflammation (n = 8). Groups are labeled as in legends of Fig. 2. White arrows indicate areas with clear infiltrated inflammatory cells. Data were shown as mean ± SEM (*p < 0.05; **p < 0.01; ***p < 0.001).

Because Suhuang inhibited inflammatory cell recruitment into the BALF, we next examined the pulmonary pathology stained with H&E. In OVA-induced asthmatic lung tissue, we observed a marked infiltration of inflammatory cells into perivascular and peribronchial connective tissues compared with mice treated with saline. Suhuang treatment at either dose significantly reduced the inflammatory cell infiltration (Fig. 4A). A semi-quantitative analysis further revealed that the inflammation was significantly decreased in the Suhuang treated groups compared with OVA group (Fig. 4B).

### Suhuang decreased mucus production and collagen fiber deposition

**Figure 5:**
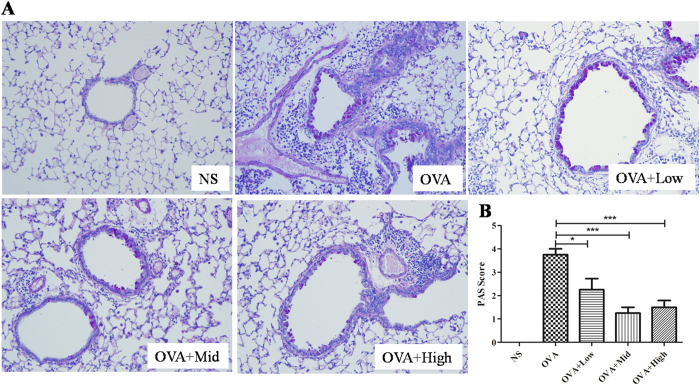
Suhuang decreases the mucus production in OVA challenge mice. (**A**) Mucus production of lung sections with PAS staining. (**B**) PAS score of each groups with semi-quantification (score: 0–4) under Olympus microscope (10 × 20 magnification). Groups are labeled as in legends of Fig. 2. White arrows indicate areas with mucus production. Data were shown as means ± SEM. (*p < 0.05; ***p < 0.01).

**Figure 6:**
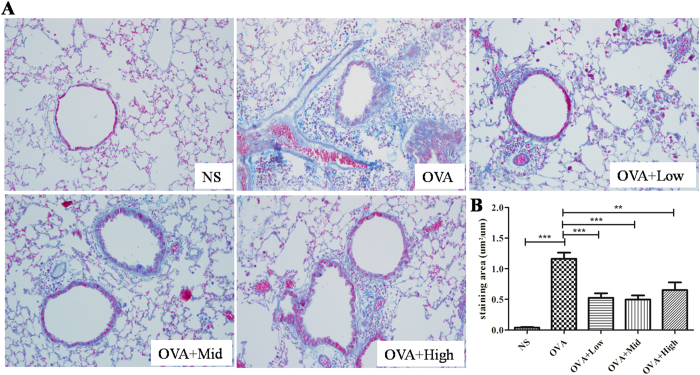
Effect of Suhuang on collagen deposition in chronic OVA challenge asthma model. (**A**) Representive images of Masson staining of lung sections. Blue areas indicate collagen deposition. (**B**) Semi-quantification of collagen deposition under Olympus microscope (10 × 20 magnification). Groups are labeled as in legends of Fig. 2. Data were shown as mean ± SEM (*p < 0.05; **p<0.01; ***p < 0.001).

Mucus hyper-secretion is another common pathophysiological feature in asthma, which might cause mortality in most severe situations. As shown in Fig. 5A, we observed a marked mucus production in OVA-induced asthmatic lung tissue, which was significantly attenuated by either dose of Suhuang. The semi-quantified results revealed that Suhuang, especially at middle and high doses, could dramatically alleviate the mucus production (Fig. 5B). Extended OVA challenge caused significant airway remodeling, which is characterized by collagen deposition, airway smooth muscle and goblet cell hyperplasia, and TGF-β1 production^14^,^15^. To examine the airway remodeling induced by chronic OVA exposure, we detected the collagen deposition using Masson trichrome staining (Fig. 6A). There was very little collagen deposition around airway walls and blood vessels in the normal mice, while it was markedly increased in the chronic asthma model. Suhuang treatment at either dose effectively reduced the OVA-induced collagen deposition after analysis under image-processing system (Fig. 6B).

### Suhuang attenuated airway remodeling associated factors IL-13 and TGF-β1

**Figure 7:**
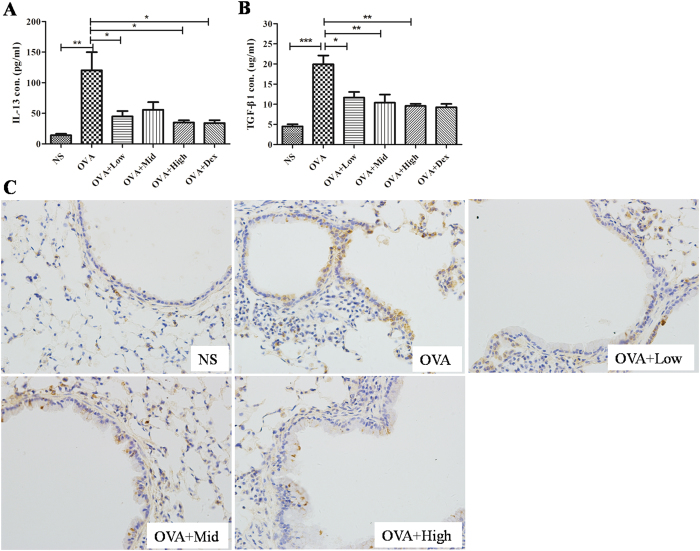
Suhuang decreases the levels of IL-13 and TGF-β1 in lung tissues. (**A,B**) Levels of IL-13 (A) and TGF-β1 (B) in lung homogenates. (**C**) Immunihistochemistry staining of TGF-β1 in lung sections. Groups are labeled as in legends of Fig. 2. Data were shown as mean ± SEM (*p<0.05; **p < 0.01; ***p < 0.001).

It has been well accepted that IL-13 is the Th2 cytokine predominately regulating mucus production and airway remodeling^16^,^17^,^18^. Also, TGF-β1 has been shown to be widely involved in tissue remodeling^15^,^19^. We next examined the effect of Suhuang on these factors and other related cytokines such as IFN-γ, IL-4, IL-5, and IL-17A in BALF and lung tissues. In lung homogenates, IL-13 was significantly increased by OVA challenge, and it was remarkably decreased in Suhuang treated groups (Fig. 7A). Similarly, TGF-β1 level in lung tissues was significantly increased by OVA challenge, which again was attenuated by Suhuang (Fig. 7B). The immunohistochemistry staining of TGF-β1 revealed that this growth factor was predominantly produced in airway epithelial cells (Fig. 7C).

The levels of IL-5 and IFN-γ in lung homogenates were not affected by chronic OVA challenge, nor modulated by Suhuang (Supplementary Figure 1). In the lungs, IL-4 was induced in the OVA group but Suhuang only slightly decreased it; IL-17A was also not significantly increased by OVA challenge, and showed mild reduction in suhuang groups as well. All these factors, including IL-13 and TGF-β1, were insignificantly changed in BALF (Supplementary Figure 2). Moreover, we examined the Th-related cytokine expression by Q-PCR in the peribronchial mediastinal lymph nodes (PMLN). IL-13 was significantly up-regulated in OVA mice, and was decreased in the Suhuang groups, while IFN-γ was down-regulated in all OVA groups. IL-17A was only slightly increased in OVA group and was not significantly affected by Suhuang (Supplementary Figure 3). These results were in consistent with the cytokine expression in the lungs.

## Discussion

Asthma, a major chronic inflammatory disease of the airways, occurs in people of all ages. According to Global Asthma Report 2014, the most recent revised global estimate of asthma suggests that as many as 334 million people have suffered this disease, and that the burden of disability is high. To date, a variety of drugs have been developed to treat asthma. Many patients worry about the side effects of conventional medical treatments for asthma, particularly ICS treatment. Therefore, compliance to current therapies is frequently poor^9^. Moreover, ICS treatment has a problem that usage might cause invalidation and waste, and exert side effects in certain patients. Capsule is a convenient manner especially for severe and elderly patients.

Hence, we aimed to detect whether Suhuang, which shows efficacy on CVA, has potential application in asthma. According to the dose used in experimental animals before clinical application, we investigated 3 doses of Suhuang that might be effective in mice to perform this experiment. Our study clearly demonstrated for the first time that Suhuang effectively prevented AHR, inflammation, and airway remodeling in a mouse model of chronic asthma. Moreover, there were not any appreciable differences between the protective effects of the two lower doses of Suhuang used throughout the study, suggesting that this drug might be used at a lower dose for treating allergic patients to reduce its possible side effects. It might be interesting to note that the high dose of Suhuang failed to reduce AHR and number of macrophages in BALF. This might be exactly the side effects due to a relative high dose of this patent drug.

AHR and mucus hyper-secretion are main pathological markers of fundamental characteristic of asthma^4^,^20^,^21^, which contributes to morbidity and mortality. Markedly upregulated production of muc5ac together with stimulated secretion leads to airflow obstruction in asthma^22^. IL-13 was reported to be the crucial mediator in eosinophil induced airway AHR and mucus production^16^,^23^,^24^. Previous studies including our investigations found that IL-13 down-regulation remit airway inflammation and AHR, as well as mucus production^25^,^26^. In our present study, we observed a significantly decreased IL-13 level in lung homogenates of Suhuang treatment groups, which should contribute to the overall protective effects in the asthma model.

Macrophages are critical component of the primary innate immune response and they are the most abundant immune cell type present under homoeostatic conditions in the lungs^27^. Based on human and animal studies, macrophages have also been found to regulate pro- and anti-inflammatory responses in the airways, suggesting that these cells have a critical role in asthma^28^. The subpopulation of macrophages located in alveoli lumen is called alveolar macrophages (AM). In our study, AHR of low and middle dose groups showed better improvement than the high dose group, which might result from that high dose of Suhuang induced a large population of macrophages in the lungs. Moreover, different subtypes of AM (i.e., M1 or M2) may exert different roles in asthma pathogenesis, including AHR^29^,^30^. We observed that high dose of Suhuang induced macrophage accumulation in the lungs, which might contribute to the increased AHR, although the phenotypes of these macrophages need to be examined.

TGF-β1 is one member of the family of structurally related growth factors expressed in a variety of lung cells such as alveolar macrophage, fiber archeocyte, epithelium, endothelium, and airway smooth muscle cells (ASMCs). TGF-β1 induces the production and release of vascular endothelial cell growth factor and plasminogen activator inhibitor, contributing to the vascular remodeling in the asthmatic airway^15^. TGF-β1 also induces proliferation, survival and extracellular matrix (ECM) secretion in ASMCs, suggesting a possible cause of increased thickness of airway tissues^15^. Inhibition of TGF-β1 including with traditional Chinese medicine could improve asthmatic symptom^26^,^31^,^32^,^33^. We demonstrated that Suhuang effectively attenuated TGF-β1 expression induced by chronic allergen exposure. Targeted therapy to IL-13 was also reported to be effective in remitting airway remodeling through reduced TGF-β1^34^. Therefore, the inhibition of TGF-β1 by Suhuang could eventually prevent subsequent collagen deposition and airway remodeling.

It is noteworthy that Th1-related cytokine IFN-γ and Th17-related cytokine IL-17A were not significantly induced in our model, nor affected by Suhuang. Although it was not clear why these cytokines were not induced by chronic OVA exposure, our data suggested that IL-13 producing Th2 response should play an important role in this study. Moreover, IL-13 and TGF-β1 in the current experimental model may originate from different cells other than lymphocytes. TGF-β1 could be produced by a variety of cells including epithelial cells, endothelial cells, fibroblasts, and granulocytes^3^. Also, it has been reported that lung epithelial cells could produce IL-13^35^. Thus, our data suggested that Suhuang might directly protect local airway injury to produce less IL-13 and TGF-β1 in addition to attenuate the allergic Th2 response.

As Suhuang is a mixture of a number of herbs, it is unlikely to demonstrate which exact compounds contribute most to the protective effect of Suhuang in asthma pathogenesis. Nevertheless, some herbs in Suhuang, especially *herba ephedrae*, have previously been shown to exert anti-inflammatory effects in various animal models or *in vitro*. For example, *Herba ephedrae*-composed San’ao decoction has been shown to inhibit asthmatic inflammation in mice, probably through an up-regulation of regulatory T cells^36^. In human airway epithelial cells, this San’ao decoction could attenuate IL-4-induced eotaxin expression^37^, which theoretically will reduce the eosinophilic inflammation *in vivo*. In guinea pigs, *herba ephedrae* extract reduced the number of citric acid-induced laryngeal cough^38^. Thus, the overall protective effect of Suhuang in asthma pathogenesis should be contributed at least in part by *herba ephedrae*. However, Suhuang should apparently have its advantages than a single herb *in vivo*, as in general, the multiple mixture medication should be more dialectical, more conciliatory, and more balanceable.

In summary, the current study demonstrated that a traditional Chinese medication Suhuang could effectively decrease OVA-induced AHR, eosinophilic airway inflammation, mucus overproduction and airway remodeling, most likely through down-regulation of IL-13 and TGF-β1. Our findings support a possible application of Suhuang as a therapeutic drug for patients with allergic asthma.

## Materials and Methods

### Preparation of Suhuang

Suhuang antitussive capsule is currently a commercial compound preparation made by Yangtze River Pharmaceutical Group Beijing Haiyan Pharmaceutical Co., Ltd. (Beijing, China). It is comprised of 9 traditional Chinese herbs: *Folium perillae* (Zisuye), the dried leaf of Perilla frutescens (L.) Britt.; *Herba ephedrae* (Mahuang), the dried rhizome of Ephedra equisetina Bge.; *Pheretima* (Dilong), the dried body of Pheretima aspergillum (E. Perrier).; *Periostracum cicadae* (Chantui), the exuviated shell of Cryptotympana pustulata Fabricius at emergence; *Fructus arctii* (Niubangzi), the dried mature seeds of Arctium lappa L.; *Fructus schisandrae chinensis* (Wuweizi), the dried mature seeds of Schisandra chinensis (Turcz.) Baill. et Wils.; *Folium eriobotryae* (Pipaye), the dried leaf of Eriobotrya japonica (Thunb.) Lindl.; *Radix peucedani* (Qianhu), the dried roots of Peucedanum praeruptorum Dunn.; *Fructus perillae* (Zisuzi), the dried mature seeds of Perilla frutescens (L.) Britt. Suhuang (0.45 g/capsule, equals to 4.5 g crude drug) was made to be 1 g/ml solution with sterile water before treating animals. The experimental doses for mice were 3.5 (low), 7.0 (middle), and 14.0 g/kg (high) with crude drug. Each group was given at an equal volume of 0.35 ml mixture of water and Suhuang solution.

### Reagents

Suhuang was kindly provided by Yangtze River Pharmaceutical Group. Dexamethasone was obtained from the second affiliated hospital of Zhejiang University (Hangzhou, China). Ovalbumin (OVA) and methacholine (Sigma Aldrich, Co., St Louis, USA), Imject alum Adjuvant (Thermo scientific, Rockford, IL. USA), and Wright-Giemsa staining (Baso Diagnostic inc. Zhuhai, China) were well prepared for the research. RNAiso Plus and RT Reagent Kit were purchased from Takara (Takara, Japan). 12-myristate 13-acetate (PMA) and ionomycin were purchased form ebioscience (ebioscience, USA).

### Experimental animals

A total of 48 male BALB/c mice (6–8 weeks) were obtained from the Slac Laboratory Animal Co. Ltd (Shanghai, China). Mice were maintained in an animal facility under standard laboratory conditions for 1 week prior to experiments. All the animal experiments were strictly conducted in accordance with the protocols approved by Ethics Committee for Animal Studies at Zhejiang University, China.

### Sensitization and OVA-challenge

All mice were randomly separated into 6 groups as follows (n = 8): control group, OVA-asthma group, Suhuang treatment groups (low dose: 3.5 g/kg, middle dose: 7.0 g/kg, high dose: 14.0 g/kg), and dexamethasone group (2.5 mg/kg), Sensitization, challenge and treatment protocols for the different groups in this study are summarized in (Fig. 1). Mice for asthma model were sensitized on day 0 and day 14 by intraperitoneal (ip.) injection of 80 ug OVA in 0.1ml and equal volume of aluminum hydroxide. 10 days after the second sensitization, mice were challenged with 1.5% OVA-NS, control group with NS instead, for 45 min for 18 days. 1 hour before each challenge, Suhuang groups accepted treatment (3.5 g/kg, 7.0 g/kg, 14.0 g/kg) through introgastric administration (ig.) and dexamethasone group accepted 2.5 mg/kg through introperitoneally (ip.).

### Lymphocyte isolation from lymph nodes and stimulation

Peribronchial mediastinal lymph nodes (PMLN) were obtained from mice of each group. Single-cell suspensions were collected by gently mincing these lymph nodes using the plunger of a 2.5 ml sterile syringe in a 45 um cell strainer. To activate the Th cells, cells were cultured in 1640 (10% FBS) and stimulated with 25 ng/ml PMA, 1 ug/ml ionomycin for 5–6 hours at 37 °C, 5% CO_2_.

### RNA isolation and Quantitative Real Time PCR analysis

RNA from PMLN cells after stimulation was extracted using RNAiso Plus (Takara, Japan) according to manufactures’ protocol. 1ug RNA was reversed transcribed to cDNA in 20 ul system by the PrimeScript RT Reagent Kit (Takara, Japan). Real-time PCR was performed using SYBR Premix Ex Taq^TM^ (Takara, Japan). The specific mouse primer sequences for RNA amplication were as described as follows:

β-actin Forward: 5′- GGCTGTATTCCCC-TCCATCG-3′,

β-actin Reverse: 5′- CCAGTTGGTAACAATGCCATGT-3′;

IFN-γ Forward: 5′- ACAGCAAGGCGAAAAAGGATG-3′,

IFN-γ Reverse: 5′- TGGTGGACCACTCGGATGA-3′;

IL-13 Forward: 5′- CCTGGCTCTTGCTTGCCTT-3′,

IL-13 Reverse: 5′- GGTCTTGTGTGATGTTGCTCA-3′;

IL-17A Forward: 5′- TCAGCGTGTCCAAACACTGAG-3′,

IL-17A Reverse: 5′- CGCCAAGGGAGTTAAAGACTT-3′.

### Assessment of AHR in response to methacholine challenge

Curative effects of suhuang on airway AHR was estimated with a FinePointe series RC system (Buxco, Electronics Inc. USA) according to the previous papers. Mice were anesthetized with 1.5% pentobarbital sodium and intubated to place in a whole body plethysmography chamber, respiratory rate (RR), airway resistance and compliance were collected every 2 seconds. Baseline of increased resistance index (RI) was determined by PBS and dose-dependent Mch (1, 2, 4, 8, 16 mg/ml) was nebulized in 10 ul. Changes in RI reflecting the airway resistance were calculated for each MCh concentration.

### Collection of BALF and differential cell count

Mice were sacrificed using an overdose of 1.5% pentobarbital sodium 24 h after the last challenge, and tracheotomy was performed. Three successive aspirations PBS (0.4 ml) was filled into left lung to collect a total 1ml volume of BAL fluid which was then resuspended well for quantity. After cell counting, BALF was centrifuged at 6000 rpm, 4 °C, for 5 min to obtain inflammatory cells. BALF supernatant was frozen at -80 °C for cytokines analysis. For cell differentiation, a total of 2–5 × 10^4^ BALF cells were placed on a slide and centrifuged (750 rpm, 2 min) to fix on, and centrifuged using a cytospin machine (StatSpin, Inc. Norwood, MA, USA). After the slides were dried, cells were stained using Wright-Giemsa buffer according to the manufacturer’s instructions.

### Preparation of lung homogenates

Lung tissue samples were weighed and homogenized in RIPA lysis buffer (100 mg tissue per milliliter) on ice. The homogenates were then centrifuged at 12000 rpm, 4 °C for 12 min. The supernatant was collected for the Elisa measurement of cytokines.

### Histological assessment of lung tissue

The left lungs were fixed in 4% buffered formalin. The fixed tissues were embedded in paraffin and the sections were stained with hematoxylin/eosin (H&E), periodic acid-Schiff (PAS) and Masson trichrome staining. The H&E staining sections were semi-quantitated (score: 0–4) as previously described for the inflammatory situation^39^,^40^. Collagen area on the basal membrane of airway was analyzed by Leica-Qwin image-processing system (Leica Imaging Systems, Bensheim, Germany). The result was expressed as collagen staining area of per micrometer length of basement membrane of bronchioles. PAS score was assessed according to the former paper: positive goblet cells in each airway were determined as follows: 0:  <5% goblet cells; 1: 5–25%; 2: 25–50%; 3: 50–75%; 4: >75%^39^,^41^. All slides were examined in a random blinded fashion by 2 independent investigators.

### Analysis of cytokines

IFN-γ, IL-4, IL-5, IL-13, IL-17A and TGF-β1 levels in BALF as well as in lung homogenates were measured by specific Elisa kits (R&D Systems, Abingdon Oxon, UK) according to the manufacturer’s instructions. The sensitivity of these assays are respectively 2 pg/ml (IFN-γ, IL-4), 7 pg/ml (IL-5), 1.5 pg/ml (IL-13), 5 pg/ml (IL-17A), 4.6 pg/ml (TGF-β1).

### Statistical analysis

One-way analysis of variance (ANOVA) was used to analyze the statistical differences among the groups, with P values indicated in the related graphs. All data are expressed as mean ± SEM. The analyses and graphs were performed using GraphPad Prism 5.0 software (GraphPad Software Inc., San Diego, CA, USA). The level of statistical significant was set at a p-value <0.05.

## Additional Information

**How to cite this article**: Zhang, C. *et al.* Suhuang antitussive capsule at lower doses attenuates airway hyperresponsiveness, inflammation, and remodeling in a murine model of chronic asthma. *Sci. Rep.* **6**, 21515; doi: 10.1038/srep21515 (2016).

## Supplementary Material

Supplementary Information
