# Supplementary material for: Suhuang antitussive capsule at lower doses attenuates airway hyperresponsiveness, inflammation, and remodeling in a murine model of chronic asthma
Source: Sci Rep. 2016 Feb 10;6:21515. doi: 10.1038/srep21515 (PMC4748281; doi:10.1038/srep21515)
Supplement: Supplementary Information [file srep21515-s1.pdf]

Supplementary information

Suhuang antitussive capsule at lower doses attenuates airway hyperresponsiveness, inflammation, and remodeling in a murine model of chronic asthma

Chao Zhang[1], Lanhong Zhang[1], Yinfang Wu[1], Tianwen Lai[1], Haisheng Wang[2], Hui Xiao[2], Luanqing Che[1], Songmin Ying[1], Wen Li[1], Zhihua Chen[1], Huahao Shen[1,3]

1. Department of Respiratory and Critical Care Medicine, Second Affiliated Hospital, Institute of Respiratory Diseases, Zhejiang University School of Medicine, Hangzhou, China.
2. Yangtze River Pharmaceutical Group Beijing Haiyan Pharmaceutical Co., Ltd, Beijing, China.
3. State Key Lab of Respiratory Disease, Guangzhou, China.

Correspondence and requests for materials should be addressed to Huahao Shen (huahaoshen@163.com) or Zhihua Chen (zhihuachen2010@163.com).

Supplementary Figure 1

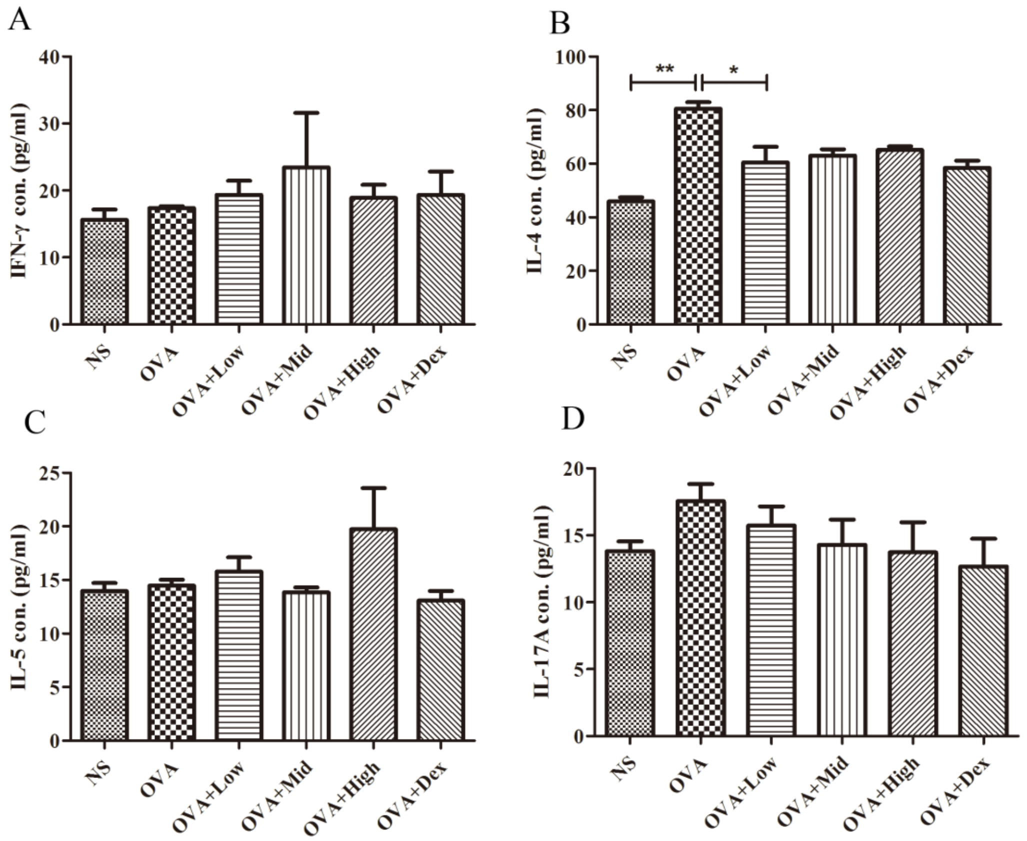

Supplementary Figure 2

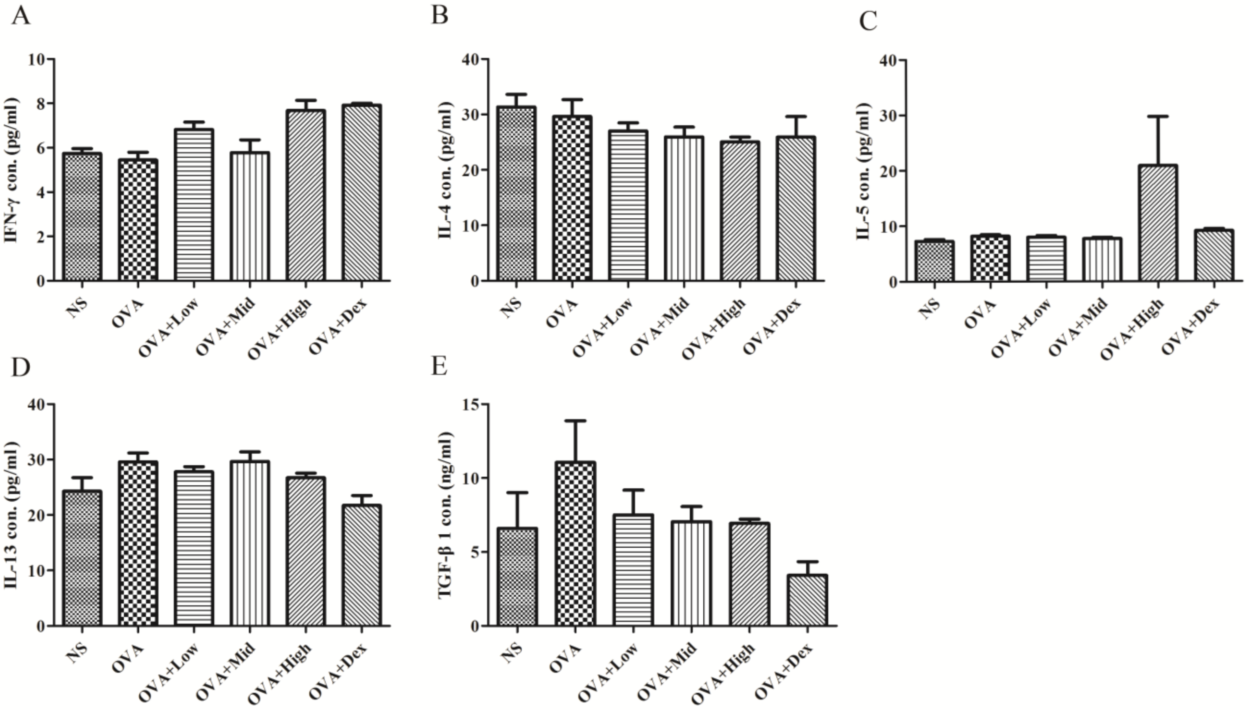

Supplementary Figure 3

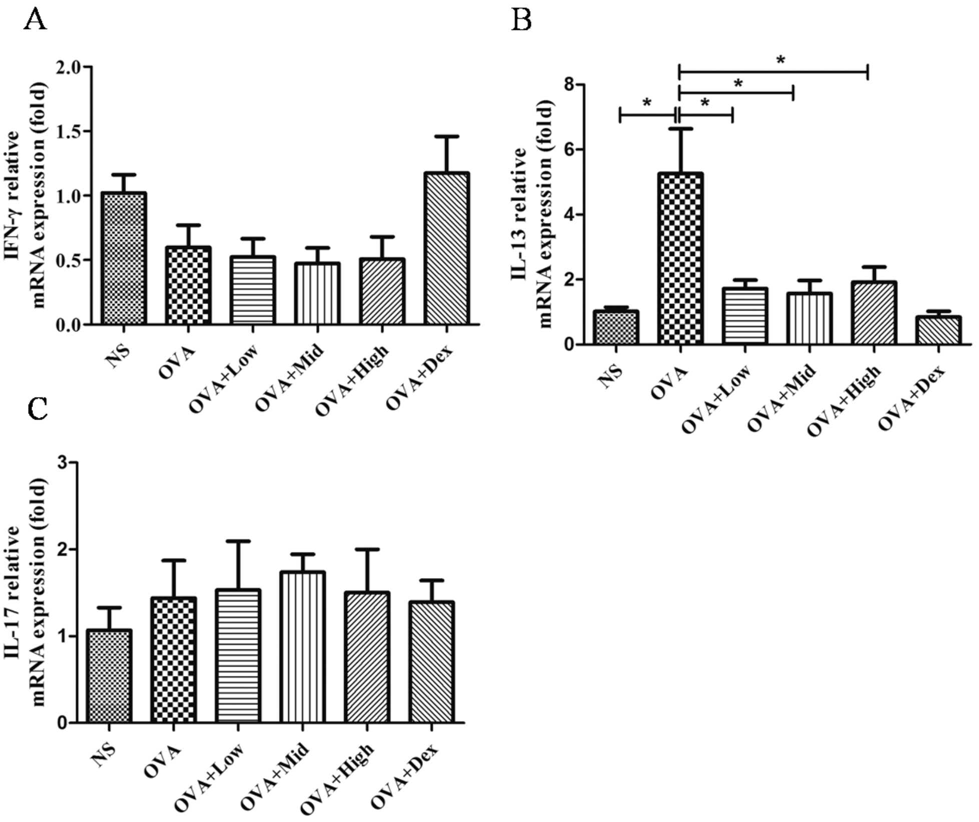

Figure legends

Supplementary Figure 1 Th1/Th2 related cytokines in lung homogenate of each group (Elisa). NS: saline controls; OVA + Low: OVA challenge and treated with 3.5 g/kg Suhuang; OVA + Mid: OVA challenge and treated with 7.0 g/kg Suhuang; OVA + High: OVA challenge and treated with 14.0 g/kg Suhuang; OVA + Dex: OVA challenge and treated with 2.5 mg/ml dexamethasone. Data were shown as mean ± SEM.

Supplementary Figure 2 Th1/Th2 related cytokines and TGF-β1 in BALF of each group (Elisa). Groups are labeled as in legends of Supplementary figure 1. Data were shown as mean ± SEM.

Supplementary Figure 3 IFN-γ, IL-13 and IL-17A mRNA expression in PMLN. Groups are labeled as in legends of Supplementary Figure 1 (*$p<0.05$).
